# *Salmonella enterica* employs metabolic adaptation to plant environments

**DOI:** 10.3389/fpls.2025.1636330

**Published:** 2025-09-08

**Authors:** Min Han, Yongming Duan, Adam Schikora

**Affiliations:** ^1^ Julius Kühn Institute (JKI) - Federal Research Centre for Cultivated Plants, Institute for Epidemiology and Pathogen Diagnostics, Braunschweig, Germany; ^2^ China National Center for Food Safety Risk Assessment (CFSA), Beijing, China; ^3^ Nutrition & Health Research Institute, COFCO Corporation, Beijing, China

**Keywords:** *Salmonella enterica*, metabolism, carbon, amino acids, adaptation to plants

## Abstract

Plant environments are considered reservoirs for *Salmonella enterica.* While exploring *Salmonella*’s adaptation mechanisms to plant environments, metabolic regulation has frequently gained attention. However, these findings have never been summarized or discussed. This review focuses therefore, on the metabolic adaptations employed by *S. enterica* to adapt to plant environments, including nutrient availability, acquisition, and its pathway regulation. Plant environments provide diverse carbon sources (e.g. sugars, organic acids, glycerol, and fatty acids) and amino acids, while *S. enterica* dynamically reprograms its metabolism to prioritize glucose via glycolysis, activate gluconeogenesis under sugar limitation, and utilize alternative carbon sources including glycerol or fatty acids. Amino acid biosynthesis, notably cysteine, also seemed critical in *S. enterica* adaptation to plant environments. These adaptive mechanisms highlight how *S. enterica* balances biosynthesis and catabolism of diverse nutrients in plant environments, offering insights into its metabolic plasticity as an adaptive strategy in agricultural ecosystems.

## 1 Introduction

*Salmonella enterica* is a bacterium adapted to animals, causing systemic or local infection in potential animal hosts (EFSA, 2025). Multiple bacterial factors contribute to infection, including Type III Secretion Systems (T3SSs) and effectors encoded by multiple *Salmonella* Pathogenicity Islands (SPIs) (Jennings et al., 2017; Lou et al., 2019). In addition to animal hosts, agricultural environments such as soil and plants can serve as potential reservoirs for *S. enterica* (Schierstaedt et al., 2019), increasing the risk of human infection. However, current knowledge of *S. enterica* adaptation mechanism to agricultural environments remains fragmented.

Microorganisms associated with plants may employ a metabolic adaptation strategy to hosts (Sit et al., 2015; Prusky and Wilson, 2018; Zhang et al., 2018), since appropriate metabolism determines their ability to persist and their potential proliferation. Generally, nutrient suitable for bacteria are widely distributed in plant tissues (e.g. xylem, phloem, and mesophyll or parenchyma cells) and ecological niches associated with plants (e.g. rhizosphere and phyllosphere) (Fatima and Senthil-Kumar, 2015). For example, *Pseudomonas fluorescens* colonization decreased the abundance of sugars in bean leaves, while the population of *P. fluorescens* grew (Mercier and Lindow, 2000), indicating that leaf-sourced sugars were consumed. The composition and population size of the associated microbiome can be altered when the nutrients supply changes (Mercier and Lindow, 2000), demonstrating the importance of available nutrients for microbiome.

*S*. *enterica* may exhibit diverse living statuses in plant-related environments, such as population increase, stability, or decline. Thus, descriptions including adaptation (Ferelli et al., 2020; Han et al., 2020), fitness (Zaragoza et al., 2012; Dixon et al., 2022), survival (Van der Linden et al., 2013; Xylia et al., 2022), persistence (Oblessuc and Melotto, 2020; Jacob et al., 2021), growth (Nugent et al., 2015; Potnis et al., 2015), colonization (Visconti et al., 2022; Dixon et al., 2024), and others have been used in previous reports. Instead of distinguishing between these varied terms, this review employs “adaptation” as an overarching concept to represent all such descriptions, focusing on bacterial population’s outcome in plant environments. Metabolic adaptation is observed in *S. enterica* for the extracellular enteric lumens (Harvey et al., 2011; Spiga et al., 2017; Herrero-Fresno and Olsen, 2018; Taylor and Winter, 2020) and *Salmonella*-containing vacuole (SCV) in cells (Dandekar et al., 2012, 2014; Taylor and Winter, 2020). Interestingly, approximately 40 - 50% of the regulated genes were overlapping in *S. enterica* infecting mice and colonizing tomato fruits, of which most were metabolism-related (de Moraes et al., 2017), showing that *S. enterica* may use metabolic adaptation strategy in plants, as well. This hypothesis was supported by several other reports. Using proteome, Kwan et al. (2015) found that more than half of the proteins extracted from *S*. *enterica* serovar Typhimurium (*S.* Typhimurium) 14028s inoculated to alfalfa sprouts seedlings were metabolism-related. On lettuce, *Salmonella*’s variable adaptation to root exudates of various cultivars (Klerks et al., 2007) and to leaves at different ages (Brandl and Amundson, 2008) was attributable in part to the content and abundance of available compounds. Similarly, the differential adaptation of *S. enterica* in apoplast of lettuce leaves was partially due to their variable nutrient utilization abilities in apoplast (Jacob et al., 2024). The improved colonization of *S*. Typhimurium SL1344 on lettuce and cilantro leaves was due to the active metabolism of carbohydrates and amino acids, the abundance of which increased with *Dickeya dadantii* co-infection (Goudeau et al., 2013). Jacob et al. (2021) reported that bacterial metabolic changes within the initial hours of *S*. *enterica* interaction with lettuce leaves were crucial for their adaptation. Using *S*. Typhimurium 14028s Transposon-Sequencing (Tn-Seq) library, we generated similar findings: genes encoding proteins in multiple metabolic pathways were necessary for *Salmonella*’s growth in tomato/lettuce leaf-mimicking media (Han et al., 2024). All these reports indicate a metabolic adaptation strategy employed by *S. enterica* in plant environments. Interestingly, there exist similarities and differences in the reprogrammed metabolic network between *S. enterica* adapting to plants or animals (de Moraes et al., 2017; Kwan et al., 2018). For instance, the catabolism of carbon sources, as well as the biosynthesis of amino acids and nucleotides, plays crucial roles in *S. enterica* adapting to both plants and animals (de Moraes et al., 2017; Kwan et al., 2018; Jacob et al., 2021). However, *S. enterica* employed different metabolic networks for serine while adapting to plants or animals (Kwan et al., 2018). Summarizing the reports focusing on the metabolic pathways used by *S. enterica* during adaptation to plant environments, sugars, organic acids, amino acids, and fatty acids metabolism, along with energy consumption and production, seemed to play crucial roles (Brankatschk et al., 2014; Kwan et al., 2015; de Moraes et al., 2017; Kwan et al., 2018; He et al., 2021; Jacob et al., 2021). This review will focus on the nutrients and the corresponding bacterial primary metabolism, which may aid in the understanding of the metabolic adaptation strategy used by *S. enterica*, and may contribute to the establishment of precise preventative strategies.

## 2 Nutrients available in plant-related environments

### 2.1 Sugars, organic acids, and amino acids are available in plant-related environments

**Figure 1:**
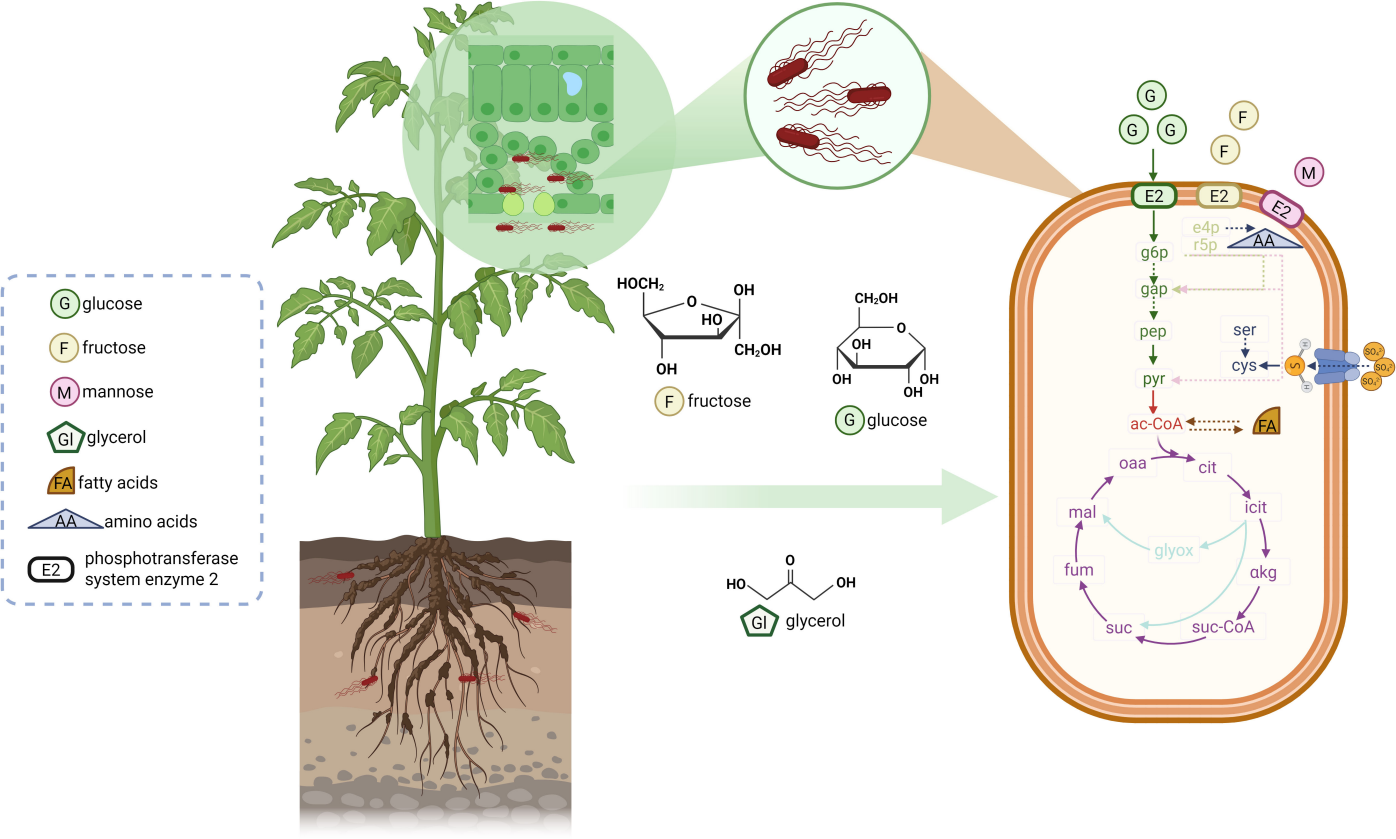
Nutrients that may be present in plant-related environments and used by *Salmonella enterica.* Plant-related environments may provide various nutrients for *Salmonella enterica*, including sugars, amino acids, organic acids, fatty acids, glycerol, and others. The varieties and abundance depend on plant species, plant growing stages, organs of plants, and other factors. Image 1 takes tomato plant as an example: structural formulas stand for glycerol as major nutrient in soil and root exudates, as well as glucose and fructose as major nutrient in leaves. *S. enterica* adapts to environments according to nutrient availability. Arrows represent metabolic pathways. Green: glycolysis; light green: pentose phosphate pathway; pink: Enter-Doudoroff pathway; red: pyruvate oxidation; purple: the tricarboxylic acid (TCA) cycle; light blue: the glyoxylate shunt. Arrows with solid and dot lines represent one step and omitted steps of reactions, respectively. Icons and legends in the dash box explain the symbols in the figure. G6p, glucose-6-phosphate; gap, glyceraldehyde-3-phosphate; pep, phosphoenolpyruvate; pyr, pyruvate; e4p, erythrose 4-phosphate r5p, ribose 5-phosphate; ser, serine; cys, cysteine; ac-CoA, acetyl-coenzyme A; cit, citrate; icit, isocitrate; αkg, α-ketoglutarate; suc-CoA, succinyl-coenzyme A; suc, succinate; fum, fumarate; mal, malate; oaa, oxaloacetate; glyox, glyoxylate. Created with BioRender.com.

Sugars, organic acids, and amino acids are major compounds available in agricultural environments, thus potentially accessible to bacteria present in those environments. Multiple sugars were detected in plant seeds, seedlings, root exudates, and leaf tissues (Lugtenberg et al., 1999; Kamilova et al., 2006; Neumann et al., 2014; Han and Micallef, 2016; Kwan et al., 2018; Jacob and Melotto, 2025). Those sugars included glucose, fructose, mannose, galactose, maltose, trehalose, ribose, sucrose, xylose, and others. Glucose and fructose were detected as the carbon sources with two highest abundances, especially in leaf tissues (Han et al., 2023a) and fruits (He et al., 2021). In matured lettuce leaves, 2.5 and 3.4 μg/g of glucose and fructose were detected, respectively (Shanmugavelan et al., 2013). In tomato fruits, glucose and fructose took up between 1.25 - 1.54% and 1.37 - 1.87% of carbohydrates, respectively (He et al., 2021). In addition to sugars, organic acids such as succinate, fumarate, and malate, which are the intermediates of the tricarboxylic acid (TCA) cycle, are detected in plants and may serve as carbon sources for bacteria (Han and Micallef, 2016; Kwan et al., 2018; Lovelace et al., 2022; Han et al., 2023a; Jacob and Melotto, 2025). As reported by Kwan et al., (2018), utilization of organic acids from germinating alfalfa seedling exudates by *S*. *enterica*, was directly measured by liquid chromatography-mass spectrometry (LC-MS). Moreover, *S. enterica* growth was associated with the abundance of organic acids in tomato plants exudates (Han and Micallef, 2016). Another report demonstrated that malate was the second most abundant metabolite in *Nicotiana benthamiana* leaves, and that the differential utilization by various *S. enterica* strains correlated with their different growth in *N. benthamiana* leaves (Lovelace et al., 2022). Interestingly, glycerol was detected as the major carbon source in diluvial sand soil, more abundant than sugars and amino acids (Carvalhais et al., 2010; Neumann et al., 2014; Prax et al., 2021; Han et al., 2023a), and may serve as a carbon source for bacteria (**Figure 1**). Glycerol was also detected in lettuce exudates (Neumann et al., 2014) and immature alfalfa seedlings (Kwan et al., 2018). In lettuce and maize root exudates (Neumann et al., 2014), as well as plant leaves, such as lettuce (Jacob and Melotto, 2025) and tomato (Han and Micallef, 2016), amino acids were detected as well. Those included alanine, aspartate, glutamate, glutamine, glycine, leucine, proline, isoleucine, serine, and threonine. In addition to the primary metabolites listed above, secondary metabolites, such as and phenolics, were detectable in tomato plants and fruits (Han and Micallef, 2016), as well lettuce leaves (Jacob and Melotto, 2025). These compounds may also serve as nutrients for bacteria. However, this manuscript focuses principally on primary metabolites from plant-related environments and the metabolic pathways utilized by *S. enterica*.

### 2.2 Factors influencing plant nutrient availability

Diversity and quantity of the available compounds vary depending on plant species, organs, developmental stages (Han and Micallef, 2016; Jacob and Melotto, 2025), and culturing substrates (especially impacting root exudates) (Neumann et al., 2014). Citrate, for example, was detected in tomato-leaf mimicking medium (Han et al., 2023a) and tomato root exudates (Kamilova et al., 2006), however, not in lettuce leaf-mimicking medium (Han et al., 2023a) nor in lettuce root exudates (Neumann et al., 2014). Tryptophan is abundant in radish and sweet pepper seedling exudates, rather than in tomato or cucumber seedling exudates (Kamilova et al., 2006). Many amino acids are scarce in alfalfa root exudates (Kwan et al., 2015), but abundant in lettuce or maize root exudates (Carvalhais et al., 2010; Neumann et al., 2014). Organic acids were differentially accumulated in lettuce leaves of diverse cultivars after being inoculated with *S. enterica* (Jacob and Melotto, 2025). Those facts suggest that the types of nutrients available for bacteria depend on are plant organ and species. In addition, plant developmental stage is also an important factor. In three weeks-old tomato seedlings, amino acids and organic acids comprised the majority of the metabolites (Han and Micallef, 2016), potentially contributing to microbiome recruitment and the establishment of stable rhizosphere or phyllosphere habitats (Qu et al., 2020). As tomato plants reached the flowering stage (six weeks-old), sugars and sugar alcohol progressively increased in proportion and became the major components (Han and Micallef, 2016), reflecting the enhanced photosynthesis abilities of plants. This shift was observed also during tomato fruit’s maturation process (Carrari et al., 2006). Another important factor in tomato phytochemical composition is the organ. Exudates from tomato fruit, shoots, and roots can differentially support the growth of *S. enterica* (Han and Micallef, 2016). All the above-mentioned reports demonstrate that plant physiological factors impact the nutrients available to bacteria.

## 3 *S. enterica* nutrient acquisition routes

In mammalian systems, nutrients in extracellular and intracellular environments are directly accessible for *S. enterica*. In agricultural environments, exudates from roots or seedlings are secreted into surrounding environments, and may be also acquired by *S. enterica* (Brankatschk et al., 2014; Kwan et al., 2015; Han and Micallef, 2016; Kwan et al., 2018). Moreover, *S. enterica* can internalize into plants via root or stomatal cavities of leaves (Jechalke et al., 2019; Zarkani et al., 2019; Chahar et al., 2021), enabling a localization within the apoplast. Leaf apoplastic fluids are abundant in sugars and organic acids (Lopez-Bucio et al., 2000; Fatima and Senthil-Kumar, 2015), providing a rich nutrient’s pool for *Salmonella*. Furthermore, plant infection by phytopathogenic bacteria, such as *Xanthomonas* spp. (Potnis et al., 2015; Cowles et al., 2022; Dixon et al., 2024) or *Pseudomonas syringae* pv. *tomato* DC3000 (Meng et al., 2013), may increase nutrient leakage from plant cells, potentially providing further nutrients for *S. enterica*. In addition, recent study reported that *S. enterica* BcsZ enzyme is responsible for its carboxymethylcellulase activity while interacting with parsley leaves (Fratty et al., 2022). This finding pointed to another potential route for *S. enterica*’s nutrient acquisition in plants. Notably, reports using plant tissue lysates to mimic plant-derived nutrients may overlook particular acquisition routes.

## 4 *S. enterica* metabolic adaptation to plants

Carbon and nitrogen availability may affect physiological status, growth, population size, and virulence of microorganisms (Mercier and Lindow, 2000; Brandl and Amundson, 2008; He et al., 2021). For example, adding sugars to the medium, where lettuce was cultured, reduced *S*. Typhimurium internalization into plants, probably because the chemotaxis directed by sugars from lettuce was misguided (Kroupitski et al., 2009). On one hand, carbon metabolic reprogramming in *S. enterica* is critical for plant adaptation. Proteomic analyses of *S*. Typhimurium inoculated to alfalfa seedling exudates revealed differential expression of sugar metabolism including glycolysis, gluconeogenesis, pentose phosphate pathway (PPP), and TCA cycle proteins (Kwan et al., 2015), a finding mirrored in *S*. Typhimurium Tn-Seq library adaptation studies using tomato/lettuce leaf-mimicking media (Han et al., 2024). On the other hand, plant’s carbon metabolic pathways are also altered during the interaction with *S. enterica*. Metabolism of glucose, fructose, sucrose, and other carbon sources was modified in lettuce leaves infiltrated with *S. enterica* (Jacob and Melotto, 2025). KEGG pathways related to carbon source metabolism were upregulated in *S. enterica* inoculated to cantaloupe and fresh tomato fruits cuts (He et al., 2021), as well as *S. enterica* inoculated into Arabidopsis leaf apoplast (Jacob et al., 2021). In contrast, infiltration of *S. enterica* into lettuce leaves induced the regulation of pathways related to multiple amino acid metabolism (Jacob et al., 2021). Similar findings on the crucial role of amino acids were also obtained in *S. enterica* adaptation to cantaloupe and fresh tomato fruits cuts (He et al., 2021), as well as tomato/lettuce leaf-mimicking media (Han et al., 2024). These reports suggest that carbon and nitrogen metabolic pathways serve as critical determinants in *S. enterica* adaptation to plant environments.

### 4.1 Carbon metabolism

#### 4.1.1 *S. enterica*’s preference for available carbon sources

Sugars are the most common carbon sources in plant-related environments. *S. enterica*’s proliferation in tomato plant and fruit exudates was positively correlated to sugar concentration (Han and Micallef, 2016). Tomato cultivars with higher levels of glucose and fructose could better support *S.* Typhimurium LT2 persistence (Han and Micallef, 2016). Although agricultural environments may include a wide range of sugars, not all sugars are utilized equally. For example, sucrose is commonly found in plants (Fatima and Senthil-Kumar, 2015), but only less than 10% of *Salmonella* strains can utilize it (Jahreis et al., 2002; Dixon et al., 2024). Instead, glucose is preferred sugar by *S. enterica*. Extracellular glucose can be imported into *Salmonella* cells via several ways, including the phosphotransferase system (PTS), transferring phosphate from phosphoenolpyruvate (PEP) to substrates. PTS consists of common PtsI (enzyme I) and HPr (*ptsH*), as well as diverse enzyme II dependent on substrates: glucose-PtsG/Crr, fructose-FruA, mannose-ManXYZ, etc. (Deutscher et al., 2006) (**Figure 1**). Genes *ptsI* and *ptsH* seemed essential for *S.* Typhimurium 14028s cultured in tomato fruit and leaf-mimicking media, as indicated by transcriptome (Zarkani et al., 2019; He et al., 2021) and Tn-Seq results (Han et al., 2024). In addition, *ptsG* and *crr* encoding glucose transporters, rather than genes encoding fructose or mannose enzyme II, were upregulated in *S.* Typhimurium 14028s grown in tomato leaf-mimicking medium (Zarkani et al., 2019), indicating that glucose, rather than fructose or mannose, was predominantly imported through the PTS. However, fructose or mannose might take precedence over other carbon sources since they can be incorporated into glycolysis.

When glucose is available, the import and utilization of other carbon sources, such as C4-dicarboxylates, may be suppressed, a phenomenon known as carbon catabolite repression (CCR) (Ullmann, 1996). Consistently, the expression *S.* Typhimurium 14028s genes encoding importers of succinate, fumarate, and malate (*dctA* and *dcuB*) (Soares-Silva et al., 2020) did not change significantly in tomato or lettuce leaf-mimicking media (Jechalke et al., 2019; Zarkani et al., 2019), where glucose was of high abundance (Han et al., 2023a). However, the growth of *S. enterica* was not impaired when genes encoding PTS glucose enzyme II were mutated by transposons (Han et al., 2024), demonstrating that alternative carbon sources could be used when glucose was inaccessible. Nevertheless, this consumption strategy may not be always very rigid. Instead of single carbon sources, *S. enterica* used sucrose and maltose as major carbon sources while adapting to cantaloupe fruit cuts (He et al., 2021). The use of multiple carbon sources occurs also in *S. enterica* infecting macrophages. In this case, both favorable glucose and unfavorable mannitol were used (Steeb et al., 2013). Therefore, the utilization of carbon sources by *S. enterica* should be evaluated based on the specific environmental context.

#### 4.1.2 Central carbon metabolism used by *S. enterica*

##### 4.1.2.1 Glycolysis and bypasses

Glycolysis is the primary glucose catabolism pathway. It is required in *S. enterica*’s adaptation to human and murine cells (Eriksson et al., 2003; Hautefort et al., 2008; Bowden et al., 2009; Gotz et al., 2010; Bowden et al., 2014; Garcia-Gutierrez et al., 2016), as wells as to plants (Zhang et al., 2014; Kwan et al., 2015; de Moraes et al., 2017, 2018; Jechalke et al., 2019; Zarkani et al., 2019; He et al., 2021; Jacob et al., 2021; Han et al., 2024). Many of the glycolysis intermediates, such as glucose-6-phosphate, 3-phosphoglycerate, 2-phosphoglycerate, or phosphoenolpyruvate were not detected in tomato/lettuce leaf-mimicking media, but were detectable in *S*. Typhimurium 14028s cultured in these media, indicating the glycolysis activity in *Salmonella* (Han et al., 2023a). Expression of *S. enterica*’s enzymes related to glycolysis was regulated when *S. enterica* was cultured in alfalfa seedling exudates (Kwan et al., 2015), within Arabidopsis and lettuce leaves (Zhang et al., 2014; Jacob et al., 2021), as well as in tomato and cantaloupe fruits (He et al., 2021). Moreover, several reports using *Salmonella* Tn-Seq library indicated that transposon insertion into glycolysis genes impaired *S. enterica* persistence or growth in plant-related environments, such as tomato fruit tissues (de Moraes et al., 2017, 2018) and tomato/lettuce leaf-mimicking media (Han et al., 2024). These results demonstrated the significance of unhindered glycolysis for carbon acquisition in *S. enterica*’s adaptation to plants. On the other hand, this deficiency in adaptation could be due to the absence of energy sources, as indicated by the function of Pgk and PykF, both involved in generation of ATP in glycolysis that acts as substrate-level phosphorylation, an important supplementary pathway for ATP generation in bacteria.

In addition, bypass via glucose-6-phophate to pentose phosphate pathway (PPP) and Enter-Doudoroff pathway (KDPGP) was observed in *S. enterica* adaptation to both mammalian cells (Lundberg et al., 1999; Eriksson et al., 2003; Hautefort et al., 2008; Gotz et al., 2010; Diacovich et al., 2017) and plant hosts (Zhang et al., 2014; Jechalke et al., 2019; Zarkani et al., 2019; Jacob et al., 2021; Han et al., 2023a). Non-detection of the enzymes catalyzing fructose-1,6-diphosphate to glycerate-1,3-phosphate in *S. enterica* internalizing into lettuce leaves might suggest the possibility of such bypasses (Zhang et al., 2014). Similarly, glucose-6-phosphate, rather than fructose-6-phosphate, was detected in *S*. Typhimurium 14028s cells grown in tomato/lettuce leaf-mimicking media (Han et al., 2023a). This bypass hypothesis was also supported by the regulation of gene expression and mutants’ adaptation. Transcriptomic analyses showed that several genes in PPP were differentially expressed in *S. enterica* infiltrated into Arabidopsis and lettuce leaves (Jacob et al., 2021). Similar results in PPP and KDPGP were also obtained in *S*. Typhimurium 14028s cultured in tomato/lettuce leaf-mimicking media compared to those cultured in minimal medium (Jechalke et al., 2019; Zarkani et al., 2019). Additionally, Tn-Seq analysis identified these genes essential for the growth of *S*. Typhimurium 14028s in leaf-mimicking media (Han et al., 2024). The occurrence of such bypass might also: i) degrade plethoric glucose-6-phosphate produced from excess glucose in leaf media, since accumulation of such phosphorylated intermediates could be toxic (Boulanger et al., 2021, 2022), and ii) form ribose 5 phosphate (r5p) and erythrose 4-phosphate (e4p) in the PPP (**Figure 1**). R5p can be converted to phosphoribosyl pyrophosphate (PRPP), an intermediary in purine and pyrimidine nucleotides biosynthesis that is essential for *S. enterica* adaptation to Arabidopsis and lettuce leaves (Jacob et al., 2021; Han et al., 2023b). Concurrently, e4p acts as the precursor of aromatic amino acids, including tryptophan, whose biosynthesis was shown vital for *Salmonella* adaptation to tomato and cantaloupe fruits (He et al., 2021). Collectively, these bypasses may assist *S. enterica* in adaptation to plant-related environments.

##### 4.1.2.2 Pyruvate oxidation

The end product of glycolysis and KDPGP is pyruvate. Pyruvate is generally converted by pyruvate dehydrogenase complex to acetyl-CoA, which serves as a bridge between glycolysis, fatty acid metabolism and the TCA cycle (**Figure 1**). In animals, *Salmonella* mutant in one of the pyruvate dehydrogenase complex encoding gene *aceE* was found less invasive to epithelial cells (Pang et al., 2011) and impaired in survival in chicken macrophages (Chang et al., 2008). Similarly, pyruvate oxidation to acetyl-CoA is required for *S. enterica* adaptation to tomato both leaf-mimicking medium and leaves (Han et al., 2023a). After infiltration, the mutant in the operon’s first gene, *aceE*, showed reduced persistence in tomato leaves, and the deficiency could be overcome by introduction of *aceEF* into the mutant (Han et al., 2023a). In addition, genes related to pyruvate metabolism pathway were enriched among genes expressed in *S. enterica* infiltrated to Arabidopsis leaves (Jacob et al., 2021). In *S. enterica* inoculated to tomato and cantaloupe fruits, several genes in the pyruvate metabolism pathway were upregulated, though the specific upregulated genes exhibited plant-dependent variation (He et al., 2021). Moreover, the *aceE* mutant had significantly reduced carbon metabolism intermediates if compared to the wild type grown in tomato-leaf mimicking medium (Han et al., 2023a), indicating that pyruvate oxidation played an important role in the regulation.

##### 4.1.2.3 The TCA cycle and shunts

One of the exits for the acetyl-CoA produced by pyruvate oxidation is the TCA cycle (**Figure 1**). A complete oxidative TCA cycle was required for *Salmonella* virulence to mice, as mutants in the TCA enzymes presented attenuated or loss of virulence to mice when the host survival was evaluated (Tchawa Yimga et al., 2006). However, conclusions on *S. enterica*’s adaptation to plants were occasionally contradictive. Expression of *sucCD* encoding succinate-CoA ligase and *sdhCDAB* encoding succinate dehydrogenase was upregulated when *S*. *enterica* serovar Weltevreden (*S*. Weltevreden) was inoculated to alfalfa sprouts (Brankatschk et al., 2014). Jacob et al. (2021) also reported differential expression of TCA cycle genes in *S. enterica* adaptation to Arabidopsis and lettuce leaf apoplast. Especially, KEGG pathway analysis revealed enrichment of the TCA cycle of *S. enterica* in Arabidopsis leaves, highlighting its critical role (Jacob et al., 2021). However, no differentially expressed enzymes of the TCA cycle were observed in *S. enterica* internalizing into lettuce (Zhang et al., 2014) or during adaptation to cantaloupe fruit fresh cuts (He et al., 2021). It was postulated by de Moraes et al. (2017) that the acetyl-CoA produced by pyruvate oxidation in *S.* Typhimurium 14028s colonizing tomato fruits was used for acetate production in the fermentation pathway rather than the TCA cycle, because mutants in *phosphate acetyltransferase* (*pta*) and *acetate kinase* (*ackA*) presented significantly reduced adaptation. In addition to the split flow of acetate, isocitrate produced in initial TCA cycle may lead towards the glyoxylate shunt (**Figure 1**). The glyoxylate shunt is typically used when sugars are insufficiently available and in order to synthesize sugars from 2C compounds such as acetate, or fatty acid degradation products. Gene upregulation of the glyoxylate shunt was observed in *S*. Typhimurium 14028s grown in soil suspension (Schierstaedt et al., 2020) and plant root exudates (Jechalke et al., 2019; Zarkani et al., 2019), where sugars are scarcely detected (Han et al., 2023a).

##### 4.1.2.4 Gluconeogenesis

Reverse to the glycolysis, gluconeogenesis is a pathway that uses energy to synthesize glucose from diverse substrates, such as pyruvate, citrate, malate, succinate, acetate, oleate, lactate, glycerol, glycogenic amino acids, and others. Several enzymes may contribute to both glycolysis and gluconeogenesis, including phosphoglucose isomerase, fructose-1,6-bisphosphate aldolase, glyceraldehyde 3-phosphate dehydrogenase, phosphoglycerate kinase, 2,3-bisphosphoglycerate-(in)dependent phosphoglycerate mutase, and enolase. Phosphoenolpyruvate (PEP) synthase (encoded by *pps*) and fructose-1,6-diphosphatase (encoded by *fbp* and *glpX*) are enzymes contributing to the irreversible steps in gluconeogenesis in *S. enterica*. The activity of gluconeogenesis enzymes is dependent on the availability of sugars, especially glucose, because its presence inhibits the activity of those enzymes (Chin et al., 1989). For example, gluconeogenesis enzyme proteins were identified in *S*. Typhimurium 14028s inoculated to alfalfa seeds (Kwan et al., 2015), where fatty acids were abundant (Hamilton and Vanderstoep, 1979). In *S*. Typhimurium 14028s inoculated to bulk soil, where sugars were not as abundant as in plant leaves (Han et al., 2023a), *pps* was upregulated (Schierstaedt et al., 2020). However, in tomato leaf-mimicking medium with abundant glucose (Han et al., 2023a), these genes were not required for *S. enterica*, as evidenced by the comparable growth between mutants and the wild-type strain (Han et al., 2024). These findings demonstrate that *S. enterica* could dynamically regulate gluconeogenic enzymes’ expression in response to carbon source availability in plant-related environments.

#### 4.1.3 Metabolism of other carbon sources by *S. enterica*

##### 4.1.3.1 Glycerol metabolism

*S. enterica* can use glycerol as a carbon source in addition to sugars and organic acids. Glycerol was detected as the major carbon source in soil (Neumann et al., 2014; Prax et al., 2021; Han et al., 2023a). Meanwhile, the KEGG pathway of glycerol metabolism was consistently enriched in lettuce leaves inoculated with *Salmonella*, irrespective of lettuce cultivars and post-inoculation time points, highlighting glycerol as a metabolite potentially mediating the bacterial-plant interaction (Jacob and Melotto, 2025). From the *Salmonella* side, the downstream product of glycerol metabolism, dihydroxyacetone, was abundant in correspondingly cultured *S*. Typhimurium 14028s cells but not in soil (Han et al., 2023a), indicating the consumption of glycerol. Moreover, the proliferation of *S*. Typhimurium LT2 has been linked to the abundance of glycerol in tomato exudates: the cultivars with richer glycerol supply, could support *Salmonella*’s proliferation better (Han and Micallef, 2016). *S*. Weltevreden adaption to alfalfa sprouts was aided by genes that contribute to the formation of glycerol-3-phospate, which could be produced *in situ* from glycerol catabolism (Brankatschk et al., 2014). Furthermore, *S*. Typhimurium SL1344 mutants deficient in glycerol uptake and catabolism, had a reduced ability to colonize alfalfa seedlings (Kwan et al., 2018), indicating that both transport and metabolism of glycerol may be important in *S. enterica* when glycerol in agricultural environments acts as the major carbon source.

##### 4.1.3.2 Fatty acid metabolism

Fatty acids are another potential carbon sources for *S. enterica*. Both biosynthesis and catabolism of fatty acids were required for *S. enterica* when it was intraperitoneally injected to mice and sampled from their spleens (Santiviago et al., 2009). However, when *S. enterica* was inoculated via the peroral route, fatty acids catabolism was not required (Tchawa Yimga et al., 2006), probably due to the restricted amount of fatty acids in the digestive tract, where diverse and abundant lipases exist. Catabolism of fatty acids is required for *S. enterica* adapting to plant-related environments when sugars are insufficient and fatty acids are available. Typical examples include immature tomato fruits (Noel et al., 2010; de Moraes et al., 2017) and exudates from germinating alfalfa seedling (older than one day) (Brankatschk et al., 2014; Kwan et al., 2018). On the other hand, lettuce leaves inoculated with *S. enterica* presented enriched fatty acid biosynthesis and degradation pathways (Jacob and Melotto, 2025). In addition to replenishing the flux of the hub compound acetyl-CoA, another driving force of fatty acid catabolism could be the degradation of medium- and long-chain fatty acids, such as palmitic acid, margaric acid, stearic acid, and oleic acid, because they can inhibit the growth of *S. enterica* (Han and Micallef, 2016). A supporting fact is that tomato fruits from cultivars with more abundant fatty acids were less conductive to support *S. enterica*’s growth (Han and Micallef, 2016).

However, in plant environments where fatty acids are scarce, fatty acid catabolism is not as important as biosynthesis (Kwan et al., 2015; de Moraes et al., 2017, 2018; Kwan et al., 2018). Shotgun proteomics detected no fatty acid catabolism proteins in *S*. Typhimurium 14028s inoculated to exudates of newly germinated alfalfa sprout (one day-old) (Kwan et al., 2015). Instead, acetyl-CoA carboxylase (encoded by *accADBC*), functioning in the initial step of fatty acid biosynthesis from acetyl-CoA, was identified (Kwan et al., 2015). *S. enterica* mutant deficient in another biosynthesis gene, *3-oxoacyl-ACP reductase* (*fabG*) displayed impaired colonization of elder alfalfa seedlings where fatty acids were available (Kwan et al., 2018), indicating that the production of fatty acids may matter in *S. enterica* even when these compounds are available in environments.

### 4.2 Amino acids metabolism

#### 4.2.1 Various amino acids are required by *S. enterica*, depending on the environment

As previously stated, amino acids can be detected in different plant-related environments. The gene cluster engaged in amino acid metabolism in *S*. Weltevreden inoculated to alfalfa sprouts, was among regulated genes (Brankatschk et al., 2014), indicating that amino acid metabolism is important for *S. enterica* grown in plant-related environments. *S. enterica* inoculated to sprouts seedlings consistently regulated expression of methionine metabolism genes 24, 48, and 96 hours post inoculation, indicating a potential role of methionine in *S. enterica* adaptive strategies (Zheng et al., 2021). In addition, *S. enterica* used histidine, glutamate, and glutamine in hydroponic alfalfa seedlings (Brankatschk et al., 2014; Kwan et al., 2015). In this case, biosynthesis, however, appeared to play a more important function than catabolism, since more biosynthesis-related enzymes were detected (Kwan et al., 2015). In addition, amino acid biosynthesis was essential for colonization of tomato, lettuce, sprouts, and broccoli (Kwan et al., 2015; de Moraes et al., 2017, 2018; Kwan et al., 2018). This is most probably owing to a scarcity of amino acids in plant-related environments. Compared to other free available nutrients, amino acids were a minor component in tomato leaves, root exudates, and fruits (Han and Micallef, 2016; Trovato et al., 2021). The concentration of all amino acids except for threonine in alfalfa seedlings is less than 70 μM (Kwan et al., 2018), tens of times lower than in mice spleens (Xiao et al., 2016; Kwan et al., 2018). Additionally, *S. enterica* consumption of amino acid during the initial days of interaction may accelerate their limitation. For instance, metabolomic analysis of lettuce leaves one day after *S. enterica* inoculation, showed a significant decrease in nine amino acids, including valine, leucine, and proline, potentially indicating their utilization by *S. enterica* (Jacob and Melotto, 2025). As a result, *de novo* biosynthesis may be required. Biosynthesis of amino acids was observed in *S.* Typhimurium 14028s when the abundance of certain amino acids, such as glycine, proline, and tryptophan, was insufficient to meet the requirement (Kwan et al., 2015). A similar phenomenon was observed in *S*. Typhimurium 14028s adapting to tomato/lettuce leaf-mimicking media. Gene Ontology terms analysis revealed that GO terms related to biosynthesis of leucine, lysine, proline, threonine, and cysteine were enriched (Han et al., 2024). However, when *S. enterica* encounters diverse environments, its amino acid metabolism may be changed accordingly. Unlike in alfalfa seedlings, biosynthesis of glutamate and glutamine were required for *S. enterica* colonizing tomato fruit via wounds (de Moraes et al., 2017, 2018), as well as for *S.* Typhimurium in low temperature-stored intact lettuce leaves (Kroupitski et al., 2013). For *S*. Typhimurium LT2 inoculated on tomato shoot and root surface, genes related to biosynthesis of tryptophan were upregulated (Han et al., 2020).

Although amino acid biosynthesis is universally reported, the fact that some amino acids can be transformed from/to other compounds complicates the link between amino acids present in environments and the corresponding bacterial adaptation. For example, the amount of glycine originating from alfalfa seedlings is far less than the *S*. Typhimurium 14028s requirement for its catabolism. Nonetheless, in the competitive index assay, growth of the mutant deficient in glycine *de novo* biosynthesis was only marginally lower than the growth of the wild type, suggesting a conversion of serine or threonine into glycine (Kwan et al., 2015).

#### 4.2.2 Cysteine biosynthesis is required for *S. enterica* adaptation to multiple environments

Among amino acids required for *S. enterica*’s adaptation to plant environments, cysteine seems to play an extraordinarily important role. Cysteine biosynthesis via sulfate assimilation serves as an important route to covert sulfur from inorganic to organic sulfur compound (Kredich and Stewart, 2008), indicating its dual significance in nitrogen and sulfur sources utilization. In addition, cysteine acts as the primary source of other organic molecules, such as glutathione, methionine, and coenzyme A (Kredich and Stewart, 2008). Cysteine residues can serve as indispensable components in the Fe-S clusters in bacterial response to environmental stresses (Wang et al., 2010). This functional importance of cysteine in *S. enterica* was frequently reported across diverse *S. enterica*-plant interactions. In *S*. Weltevreden inoculated to alfalfa sprouts, more than half of the amino acid biosynthesis regulated genes were related to cysteine acquisition and biosynthesis (Brankatschk et al., 2014). *S*. Weltevreden inoculated to lettuce and corn salad leaves exhibited a similar result (Brankatschk et al., 2014), as did *S*. Typhimurium 14028s inoculated to immature and mature tomato fruits (Noel et al., 2010). Jacob et al. (2021, 2025) reported bidirectional enrichment among regulated genes related to the cysteine metabolism pathway in *S. enterica* adaptation to lettuce leaves, demonstrating its important role as a key metabolic hub in *Salmonella-*plant interactions. *S*. Typhimurium 14028s grown in tomato/lettuce leaf-mimicking media also required cysteine biosynthesis (Han et al., 2024). Furthermore, cysteine biosynthesis was involved in the response of *S. enterica* to abiotic stressors (Wang et al., 2010). Asides from *S. enterica*, cysteine biosynthesis was necessary in other enteric and phytopathogenic bacteria adaptation to plants, such as *Escherichia coli* on lettuce leaf surface (Fink et al., 2012) and in leaf lysates (Kyle et al., 2010), as well as *Pseudomonas syringae* on bean leaves (Marco et al., 2005).

Cysteine can be synthesized in two pathways. One is the serine conversion, and related genes such as *serine acetyltransferase* (*cysE*) and *cysteine synthase A* (*cysK*) were required for *S*. Typhimurium 14028s proliferation in tomato/lettuce leaf-mimicking media (Han et al., 2024). Similarly, when inoculated to alfalfa seedlings, the *cysE* mutant of *S*. Typhimurium SL1344 displayed decreased competitiveness compared to the wild type, this could be partially complemented by adding additional cysteine (Kwan et al., 2018). Another pathway is the assimilation of sulfate as mentioned above, which could be acquired from extracellular space and presented a crucial role in *S*. Typhimurium 14028s growth in tomato/lettuce leaf-mimicking media (Han et al., 2024). Similar finding was observed in *S*. Weltevreden inoculated to alfalfa sprouts (Brankatschk et al., 2014). Furthermore, *S. enterica* survival in egg white (Liu et al., 2021) and chlorine-based oxidative stress (Wang et al., 2010) was also related to sulfate assimilation. All those reports indicate that cysteine biosynthesis plays a crucial role in *S. enterica*’s adaptation to different environments, including plants (**Figure 1**).

## 5 Conclusions and critical issues

This review summarizes the current understanding of *S. enterica*’s metabolic adaptation to plant environments, highlighting its remarkable flexibility in utilization and biosynthesis of diverse metabolites as well as in the reprogramming of metabolic networks. Agricultural ecosystems, including plants, are able to provide multiple nutrients for *S. enterica*, including sugars, organic acids, glycerol, amino acids, fatty acids, and others. Both diversity and abundance of those compounds, which fluctuate depending on plant species, organs, developmental stages, and other physiological status, affect *S. enterica*’s adaptation. Notably, in particular studies, exudates are manually collected, or plant lysates are used mimicking the nutrients availability, and this may mask the metabolites that *S. enterica* encounters in native niches. Consequently, findings on *S. enterica* metabolic adaptation should be indeed treated with caution. Current evidence, even though, primarily derived from transcriptomic, proteomic, metabolomic, and Tn-Seq analyses, has outlined key metabolic pathways. It is however, important to note that many omic-derived findings lack the validation via other methods. In addition, further bidirectional studies on both, metabolome of *S. enterica* and plant environments should provide insights into *S. enterica* metabolic adaptation to plant environments.
